# Sesamin alleviates lipid accumulation induced by elaidic acid in L02 cells through TFEB regulated autophagy

**DOI:** 10.3389/fnut.2024.1511682

**Published:** 2024-12-20

**Authors:** Xueli Liang, Tianliang Zhang, Xinyi Cheng, Hang Yuan, Ning Yang, Yanlei Yi, Xiaozhou Li, Fengxiang Zhang, Jinyue Sun, Zhenfeng Li, Xia Wang

**Affiliations:** ^1^School of Public Health, Shandong Second Medical University, Weifang, China; ^2^Experimental Center for Medical Research, Shandong Second Medical University, Weifang, China

**Keywords:** NAFLD, elaidic acid, sesamin, autophagy, mitophagy

## Abstract

**Introduction:**

Non-alcoholic fatty liver disease (NAFLD) is a common chronic disease seriously threatening human health, with limited treatment means, however. Sesamin, a common lignan in sesame seed oil, exhibits anti-inflammatory, antioxidant, and anticancer properties. Our previous studies have shown an ameliorative effect of sesamin on lipid accumulation in human hepatocellular carcinoma (HePG2) induced by oleic acid, with its protective effects unclear in the case of 9-trans-C18:1 elaidic acid (9-trans-C18,1).

**Methods:**

L02 cells, an important tool in scientific researches due to its high proliferation ability, preserved hepatocyte function, and specificity in response to exogenous factors, were incubated with 9-trans-C18:1 to establish an *in vitro* model of NAFLD in our study. The lipid accumulation in cells and the morphology of mitochondria and autolysosomes were observed by Oil Red O staining and transmission electron microscopy. The effects of sesamin on oxidative stress, apoptosis, mitochondrial function, autophagy as well as related protein levels in L02 cells were also investigated in the presence of 9-trans-C18:1.

**Results:**

The results showed that sesamin significantly accelerated the autophagy flux of L02 cells induced by 9-trans-C18:1 as well as elevated protein levels of transcription factor EB (TFEB) and its downstream target lysosome-associated membrane protein 1(LAMP1), along with up-regulated levels of TFEB and LAMP1 in the nucleus indicated by Immunofluorescence. In addition, PTEN-induced putative kinase 1 and Parkin mediated mitophagy was activated by sesamin. The direct inhibitor Eltrombopag and indirect inhibitor MHY1485 of TFEB reversed the protective effect of sesamin, suggesting the involvement of autophagy in the lipid-lowering process of sesamin.

**Discussion:**

This work suggests that sesamin regulates autophagy through TFEB to alleviate lipid accumulation in L02 cells induced by 9-trans-C18:1, providing a potential target for the prevention and treatment of NAFLD.

## 1 Introduction

Trans fatty acids (TFAs), non-conjugated unsaturated fatty acids bearing one or more trans double bonds and presenting elaidic acid (9-trans-C18:1) as the primary isomer, are mainly obtained from hydrogenated vegetable oils and ruminant milk by humans (1). Epidemiological studies exhibit close correlations between TFAs intake and cardiovascular disease, atherosclerosis, diabetes, NAFLD or other metabolic diseases (2, 3). Industrial TFAs can give rise to inflammation, endoplasmic reticulum stress, oxidative stress, and promote the preferential storage of fat in the liver, increasing the ratio of liver fat mass, steatosis, liver cholesterol levels, alanine aminotransferase activity, and fibrosis markers, indicating the enhancement of NAFLD (4).

NAFLD, a chronic liver metabolic disorder induced by abnormal lipid metabolism, includes fat infiltration in more than 5% of liver cells, non-alcoholic steatohepatitis (NASH), fibrosis, and liver cirrhosis ultimately leading to hepatocellular carcinoma, with a global prevalence of approximately 25% (5, 6). NAFLD is associated with increased risks for liver related complications as well as cardiovascular diseases (7). Oleic acid can bind with triglycerides, cholesterol esters, phospholipids, and other long-chain fatty acids, leading to lipid metabolism disorders and increasing the potential risk for NAFLD (8). However, there is still limited evidence regarding the potential association between NAFLD and TFAs. Promoting lipid metabolism has become an effective means for treating obesity and NAFLD (9). Therefore, exploring the mechanisms of action for TFAs may provide a basis for treating diseases characterized by lipid metabolism disorders.

Although some drugs have been employed for the treatment of NAFLD related metabolic disorders, they may bring some unpredictable side effects (10). As a member of the benzodioxolane family, sesamin is a fat-soluble furan lignan with molecular formula as C_20_H_18_O_6_ and molar mass as 354.35 g/mol. Sesamin is mainly derived from sesame, and has also been found in more than 30 other medicinal plants (11). Sesamin exerts cholesterol lowering effects in serum and liver, as well as anti-inflammatory and antioxidant ones, and can alleviate lipid metabolism disorders (12). Sesamin can eliminate reactive oxygen species (ROS) and free radical induced damage to mitochondria, as well as promoting fatty acid oxidation to improve fat accumulation in the body (13). Shi et al. found that sesamin up-regulates the levels of proteins related to fatty acid oxidation, cholesterol efflux, and lipid metabolism via activating the AMP-activated protein kinase (AMPK) and peroxisome proliferator-activated receptor alpha (PPARα) signaling pathways, thereby reducing intracellular lipid accumulation during NAFLD (14). In addition, sesamin can improve hepatic steatosis and fibrosis in mice with NASH (15). However, further research is needed to determine whether sesamin can inhibit TFAs induced hepatic steatosis and the potential underlying mechanisms.

Dysregulated lipid metabolism and oxidative stress are closely related to the occurrence and development of NAFLD, with autophagy playing a key role. The double membrane structure of autophagosomes isolates cytoplasmic substances and fuses with lysosomes, degrading the contents subsequently in lysosomes (16). Autophagy is necessary for regulating lipid metabolism since it could degrade lipid droplets (17). Therefore, autophagic disruption induced by steatosis may exacerbate liver lipid accumulation (18). Overexpression of liver specific autophagy related factor 7 (ATG7) induces autophagy and reduces steatosis and damage in NASH mice (19). However, the roles of sesamin in alleviating hepatic steatosis and oxidative stress, and activating autophagy has not been fully elucidated.

The transcription factor EB (TFEB) has been identified as a major functional regulator promoting autophagy and lysosomal biogenesis at the transcriptional level (20, 21). As the primary transcriptional regulator for autophagy lysosome pathway, TFEB positively regulates the expression of genes related to autophagy and lysosome biogenesis, thereby promoting autophagosome formation, autophagosome lysosome fusion, and degradation of autophagic substrates (22). TFEB also plays an important part in physiological processes such as lipid metabolism (23). Insufficient number of lysosomes and the declined hydrolytic enzyme activity are correlated with the occurrence of NAFLD (24). Upregulated TFEB can activate mitophagy and improve mitochondrial homeostasis (25). These findings emphasize the potential role of TFEB in treating NAFLD by promoting mitophagy dependent lipid degradation. However, it remains unclear whether TFEB plays a beneficial role in the regulation of hepatic autophagy flux and lipid degradation by sesamin. L02 cell line has become an important tool in scientific researches due to its high proliferation ability, preserved hepatocyte function, and specificity in response to exogenous factors. In the present work, L02 cells were employed as an *in vitro* model to investigate the alleviative effects of sesamin on steatosis induced by 9-trans-C18:1 and the underlying mechanisms (26). The results suggest that sesamin activates autophagy flux through the TFEB mediated autophagy-lysosome pathway, thereby reducing lipid accumulation.

## 2 Materials and methods

### 2.1 Materials

The Cell Counting Kit-8 (CCK-8) (MA0218, Dalian, China) was provided by Meilun Biotechnology, with eltrombopag (EO) (SB-497115) and MHY1485 (HY-B0795) purchased from MedChemExpress. The triglyceride (TG) kit (A110-1-1), trace malondialdehyde (MDA) kit (A003-1), and trace reduced glutathione (GSH) kit (A006-2-1) were provided by Nanjing Jiancheng Bioengineering Institute (Nanjing, China). Improved Oil Red O staining kit (C0158S), 4% paraformaldehyde (P0099), bicinchonininc acid (BCA) protein concentration determination kit (P0012), ROS detection kit (S0033S), adenosine triphosphate (ATP) detection kit (S0026), mitochondrial membrane potential (MMP) detection kit (C2006), mitochondrial far-infrared fluorescence probe (C1032-50ug), Mito Tracker Green (C1048), Lyso Tracker Red (C1046), Hoechst 33342 (C1027), lipid droplet green fluorescence detection kit boron-dipyrromethene (BODIPY) 493/503 (C2053S), cell autophagy staining (monodansylcadaverine (MDC) method) detection kit (C3018S), along with adenovirus expression of monomeric Cherry (mCherry)-green fluorescent protein (GFP)-microtubule-associated protein 1 light chain 3 beta (LC3B) fusion protein (Ad-mCherry GFP-LC3B) (C3011-1mL) kit were purchased from Beyotime (Shanghai, China). The apoptosis detection kit (556547) was purchased from BD (Beijing, China).

### 2.2 Cell culture and treatment

L02 cells (Shanghai Institute of Life, Chinese Academy of Sciences) were cultured in RPMI 1640 medium containing 10% fetal bovine serum (supplemented with double antibiotics and nonessential amino acids) at 37°C under 5% CO_2_. Cells in exponential growth phase (3–10 passages) were seeded into various cell culture plates at moderate density for 24 h. The adherent cells were subjected to 9-trans-C18:1 (CAS: 112–79-8, E4637, Sigma-Aldrich, St. Louis, Missouri, United States) and/or sesamin (CAS: 607–80-7, SMB00705, Sigma-Aldrich, St. Louis, Missouri, United States) for 24 h. The study was divided into control, model, and intervention groups, respectively.

### 2.3 Cell viability

After L02 cells reach 80% coverage, they were digested and seeded into a 96 well plate. After adhesion, treatment with different fatty acids for 24 h was conducted. Then 10% CCK-8 medium were added into each well, followed by incubation at 37°C for 0.5–4 h. The absorbance at 450 nm was detected using a microplate reader (Multiskan GO, Thermo Fisher Scientific, Vantaa, Finland).

### 2.4 Oil red O staining

The cultured L02 cells were fixed with 4% paraformaldehyde at room temperature for 30 min and washed twice with phosphate buffered solution (PBS), respectively. Then, the cells were subjected to an appropriate amount of staining washing solution for 20 s and Oil Red O staining working solution for 20 min successively, followed by washing with washing solution and PBS for 30 and 20 s, respectively. The cell nucleus was stained with hematoxylin for 1 min. Microscopic observations were conducted using a microscope (DMI4000B, Leica, Germany). After dissolving intracellular lipid droplets with 100% isopropanol, semiquantitative analyses were performed by measuring the absorbance at 510 nm using a microplate reader (Multiskan GO, Thermo Fisher Scientific, Vantaa, Finland).

### 2.5 Ultrastructural observation

The treated L02 cells were digested and centrifuged at 2000 × g for 15 min. Subsequently, cell precipitates were collected and fixed with 3% glutaraldehyde fixative, dehydrated, infiltrated, embedded, ultra-thin sectioned, stained, successively. Finally, the ultrastructure was characterized using a transmission electron microscopy (TEM, HT7700, Hitachi, Nakahikari, Ibaraki, Japan).

### 2.6 Measurement of intracellular TG, MDA, and GSH

The cultured and treated L02 cells were collected and washed twice with PBS. After being mixed with 300 μL PBS, the cells were sonicated in an ice water bath. The intracellular levels of TG (A110-1-1, Nanjing Jiancheng), MDA (A003-1, Nanjing Jiancheng), and GSH (A006-2-1, Nanjing Jiancheng) were detected according to the supplier’s instructions. A microplate reader (Multiskan GO, Thermo Fisher Scientific, Vantaa, Finland) was applied to measure absorbances at specific wavelengths.

### 2.7 Measurement of intracellular ATP

Intracellular ATP was quantified using a commercial reagent kit. After removing the culture medium, 200 μL of lysis buffer was added into each well of the 6-well plate to fully lyse the L02 cells. The supernatant was obtained by centrifugation at 4°C and 1.2 × 10^4^ g for 5 min. After 100 μL ATP detection working solution was added into each detection well, 20 μL sample or standard was added and mixed quickly. The absorbance was measured using a multifunctional enzyme-linked immunosorbent assay reader (SpectraMax i3x, Molecular Devices, San Jose, United States). The concentrations of ATP in the samples were calculated based on the standard curve.

### 2.8 Fluorescence staining

After the medium removal, the L02 cells were washed once with PBS and incubated with 5 μM 2′,7′-dichlordihydrofluorescein diacetate (DCF-DA),1 × 5,5′,6,6′-tetrachloro-1,1′,3,3′-tetraethylbenzimi-dazolylcarbocyanine iodide (JC-1), 200 mM Mito Tracker, and 1× MDC fluorescent probes prepared in serum-free 1640 medium at 37°C for 30 min. Then wash them once with PBS and medium, and replace with fresh medium. The intracellular ROS, MMP, mitochondria, and autophagy degrees were observed under a fluorescence microscope (DMi8-M, Leica, Germany). The semiquantitative analyses were performed using the Image J software.

### 2.9 Apoptosis

After removing the culture medium, L02 cells were washed once with pre-cooled PBS and then subjected to digestion with ethylene diamine tetraacetic acid (EDTA) free trypsin, washing, centrifuge, and resuspension in 1× Binding buffer. Each sample was added with 5 uL Annexin-V and PI in the dark and incubated at room temperature for 15 min. Then the apoptosis was analyze using a flow cytometer (FACS AriaIII, BD, United States) within 1 h.

### 2.10 Mito-Lyso immunofluorescence co-localization

Mito Tracker Green and Lyso Tracker Red were employed for the co-localization of mitochondria and lysosomes. After culture medium removal and washing once with PBS, L02 cells were added with Mito Tracker Green or Lyso Tracker Red storage solution diluted with culture medium at a ratio of 1:10000 and 1:13333, respectively, followed by incubation at 37°C for 30 min. After removing the staining solution and adding fresh culture medium, microscopic observations were carried out by a laser confocal microscope (TCS SP8, Leica, Wetzlar, Germany). Semiquantitative analyses were performed using the Image J software.

### 2.11 Ad-cherry-GFP-LC3B adenovirus transfection

After adhesion, L02 cells cultured in confocal dishes were transfected with Ad-mCherry-GFP-LC3B at a multiplicity of infection of 40 for 48 h and subsequently subjected to different treatment based on grouping. After removing old and adding fresh culture medium, they were observed using a laser confocal microscope (TCS SP8, Leica, Wetzlar, Germany). Semiquantitative analyses were performed based on yellow and red spots.

### 2.12 Quantitative PCR analyses

Total RNA was extracted from cells using TriZol reagent (15596026, Invitrogen, Carlsbad, CA, United States), followed by reverse transcription of mRNA into cDNA using a 1st strand cDNA synthesis kit (Code No. 6210A). qPCR was performed on QantStudio 1 (Applied Biosystems, Walktham, MA, United States) using a 20 μL amplification system consisted of 10 μL premix (SsoFast EvaGreen Supermix, BioRad, CA, USA), 1 μL primer for each, 1 μL cDNA, and 7 μL deionized H_2_O. The qPCR reaction conditions were set as following: 95°C 30s for 1 cycle, along with 95°C 5s, 60°C 30 s, and 72°C 30 s for 40 cycles. Subsequently, melting curve analyses were performed to avoid amplification of non-specific products. *β*-actin was employed as an internal reference gene, and the mRNA relative level of the targets were calculated using the 2^-ΔΔCt^ algorithm. The primer sequences used in this work were presented in the Table 1.

**Table 1:** Primer sequences used for qPCR in the present work.

Target	Forward primer (5′-3′)	Reverse primer (5′-3′)
LC3	GGTGAGTGTGTCCACGCCCAT	GGTGGGTTGGTGCCCTCTGAC
p62	CTGTAACCTGCTGGATGGGACTC	TGGAAGGCATTTATTTGCTTTGTC
TFEB	TGGAGGAGGGCGATGTGCTG	GCCAGACAGGCACTAAGTCCAAACA
LAMP1	AGGCGGTGAGATCTAGACGA	AAGAATAGTGTTGGCGGGGG
ACTB	CAGTCGGTTGGAGCGAGCAT	GGGACTTCCTGTAACAACGCATCTC

### 2.13 Immunofluorescence staining

After removing the culture medium, L02 cells cultured in confocal dishes were washed thrice with PBS and fixed with 4% paraformaldehyde at room temperature for 20 min successively, followed by washing thrice with PBS and 0.1% Triton X-100 addition. After being kept at room temperature for 10 min and washed thrice with PBS subsequently, they were added with protein free rapid blocking solution (PS108P, Epizyme, Shanghai, China) and blocked at room temperature for 30 min. After removing the blocking solution, an incubation overnight at 4°C with primary antibodies against TFEB (1:100, TN27339, Abmart, Shanghai, China) and lysosome-associated membrane protein 1 (LAMP1) (1:20021997-1-AP, Protein, Wuhan, China) was performed. After washing thrice with PBS (5 min for each), the cells were incubated at room temperature for 1 h in the dark with Cy3 labeled sheep anti rabbit IgG (H + L) secondary antibody (1:200, A0516, Beyotime, Shanghai, China), followed by washing thrice with PBS (5 min for each). Hoechst 33342 (C1025, Beyotime, Shanghai, China) was used for nuclear staining. Finally, microscopic observations were carried out using a laser confocal microscope (TCS SP8, Leica, Wetzlar, Germany).

### 2.14 Western blotting

The treated L02 cells were subjected to radio immunoprecipitation assay (RIPA) lysis buffer to extract total protein and a BCA assay kit for protein concentration measurement. Thirty μg protein was uploaded into each well and separate by sodium dodecyl sulfate-polyacrylamide gel electrophoresis (SDS-PAGE). Then, the proteins were transferred onto a polyvinylidene fluoride (PVDF) membrane at a constant current of 300 mA, followed by block with 5% skim milk. Then the membrane was incubated with primary antibodies against microtubule-associated protein 1 light chain 3 (LC3) (1:1000, 14600-1-AP, Proteintech, Wuhan, China), PTEN-induced putative kinase 1(PINK1) (1:1000, DF7742, Affinity Bio sciences, Changzhou, China), Parkin (1:1000, PAL060Hu01, Cloudclone, Wuhan, China), Sequestosome 1(SQSTM1/p62) (1:1000, PAD198Hu01, Cloud-clone, Wuhan, China), TFEB (1:800, TN27339, Abmart, Shanghai, China), LAMP1 (1:1000, 21997-1-AP, Protein, Wuhan, China), glyceraldehyde-3-phosphate dehydrogenase (GAPDH) (1:1000, 10494-1-AP, Proteintech, Wuhan, China), and Histone H3 (1:1000, AF0863, Affinity, Jiangsu, China) on a shaker overnight at 4°C. After being washed with tris-buffered saline with Tween 20 (TBST) (5 min for each), the membrane was incubated with HRP labeled sheep anti rabbit secondary antibody (1:2000, SA00001-2, Proteintech, Wuhan, China) at room temperature for 1 h, followed by washing with TBST thrice (5 min for each). Finally, the protein bands were visualized using an ECL chemiluminescence assay kit (P0018S, Beyotime, Shanghai, China) in a chemiluminescence imaging instrument (5200 Multi, Tanon, Shanghai, China). The ImageJ software (NIH, Bethesda, MD, United States) was employed to analyze the densities of protein bands, with GAPDH acting as the reference gene.

### 2.15 BODIPY 493/503 fluorescence staining

Lipid droplets were stained using BODIPY fluorescent probe. After removing the culture medium, L02 cells cultured in a 24 well plate were washed twice with PBS and then fixed with 4% paraformaldehyde at room temperature for 15 min, followed by rinse twice with PBS. After adding 250 μL staining solution into each well, an incubation at room temperature in the dark for 20 min was performed. Hoechst 33342 was utilized for nuclear staining. After being washed twice with PBS, the cells were observed under a laser confocal microscope (TCS SP8, Leica, Wetzlar, Germany) and semi-quantitative analyses were performed using the Image J software.

### 2.16 Statistical analyses

All data were presented as means ± standard deviation (SD) and analyzed using the SPSS software (version 22.0, SPSS Inc., Chicago, Illinois, United States). One-way ANOVA (analyzes of variance) was employed for group comparison. LSD was applied for homogeneous variance and Dunnett T3 for the non-homogeneous. When *p* < 0.05, it was considered statistically significant.

## 3 Results

### 3.1 Sesamin alleviates lipid accumulation induced by 9-trans-C18:1 in L02 cells

**Figure 1:**
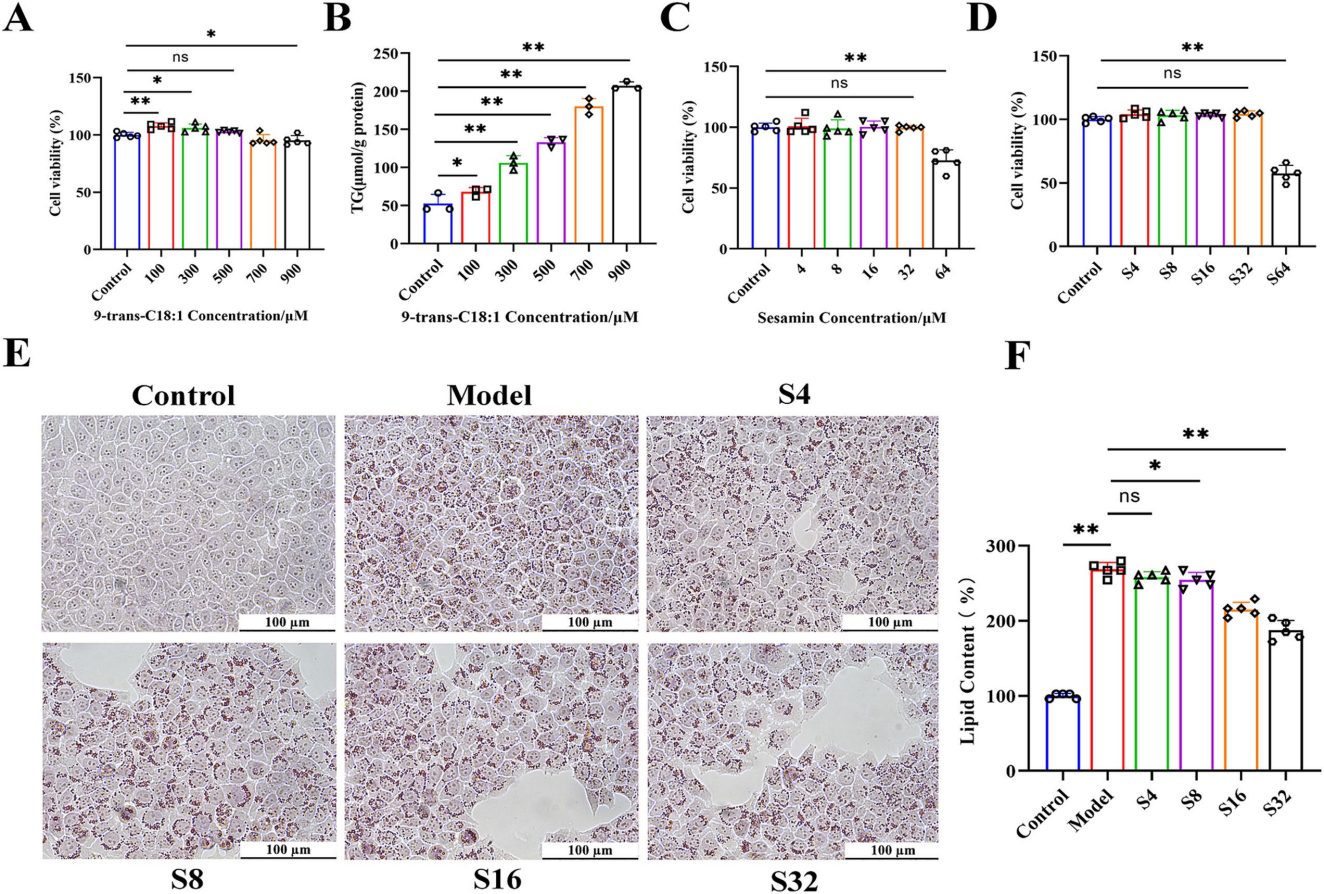
Effects of 9-trans-C18:1 and sesamin at different concentrations on cell viability and lipid accumulation in L02 cells. **(A,C,D)** cell viability detected by CCK-8 method; **(B)** Intracellular TG levels after stimulation with 9-trans-C18:1; **(E,F)** Oil Red O staining and quantitative analyses on intracellular lipid content, magnification = 400×; Scale bar = 100 μm. Control: RPMI 1640 complete culture medium; Model: 500 μM 9-trans-C18:1 + RPMI 1640; S4: 500 μM 9-trans-C18:1 + 4 μM Sesamin; S8: 500 μM 9-trans-C18:1 + 8 μM sesamin; S16: 500 μM 9-trans-C18:1 + 16 μM Sesamin; S32: 500 μM 9-trans-C18:1 + 32 μM sesamin; S64: 500 μM 9-trans-C18:1 + 64 μM sesamin. All data were represented as means ± SD (*n* = 3–5); *p < 0.05; * *p < 0.01.

L02 cells were treated with 9-trans-C18:1 at various concentrations to detect its cytotoxicity. The results of CCK-8 (Figure 1A) showed that 9-trans-C18:1 at 500 μM exerted no significant effect on cell viability, and led to significantly increased intracellular TG (Figure 1B) (*p* < 0.01), indicating the successful construction of the *in vitro* high-lipid model. In the absence or presence of 9-trans-C18:1 at 500 μM, sesamin at higher than 32 μM caused a significant decrease in cell viability (Figures 1C,D). The results of Oil Red O staining (sesamin at 4, 8, 16, and 32 μM, respectively) showed a dose-dependent decrease in intracellular lipid accumulation (Figure 1E). Therefore, 32 μM was selected as the working concentration of sesamin for subsequent experiments. These data indicate that sesamin reduced lipid accumulation in L02 cells treated with 9-trans-C18:1.

### 3.2 Sesamin restores mitochondrial homeostasis by enhancing mitophagy

**Figure 2:**
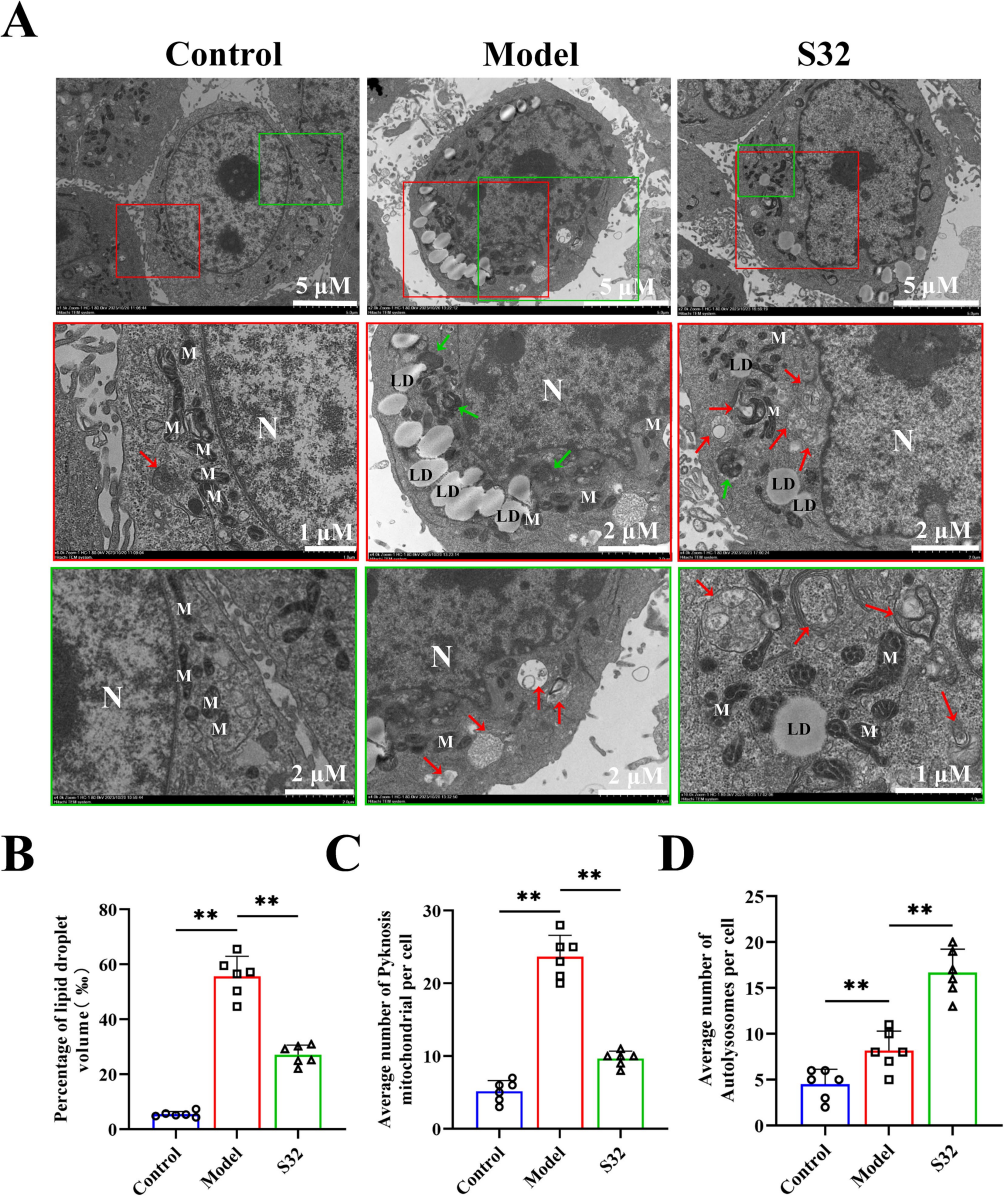
Sesamin enhances mitophagy and alleviates lipid accumulation in L02 cells. **(A)** Mitochondrial microstructure and lipid droplet accumulation observation by TEM. The red and green boxes represent enlarged images of representative parts; **(B)** Volume ratio of oil droplets in cells; **(C)** Number of pyknotic mitochondria; **(D)** Number of autophagic lysosomes; N: Nucleus; M: Mitochondria; LD: lipid droplets; The green arrow represents pyknotic mitochondria; The red arrow represents autolysosomes. Control: RPMI 1640 complete culture medium; Model: 500 μM 9-trans-C18:1 + RPMI 1640; S32: 500 μM 9-trans-C18:1 + 32 μM sesamin. All data were represented as means ± SD (*n* = 6), *p < 0.05; **p < 0.01.

The ultrastructure of L02 cells were analyzed through TEM. As shown in Figure 2, the mitochondria in control group were oval and elongated, with clear cristae structures. Compared with control, intracellular lipid droplets in model group increased significantly, with significantly changed morphology and structure of mitochondria: higher proportion of slender mitochondria and blurred cristae. Partial mitochondrial cristae structures disappeared completely, showing shrinkage necrosis and vacuolization (Green arrow). Few mitochondria were engulfed by lysosomes and exhibited characteristic autophagic lysosomes (Red arrow), indicating low levels of cellular autophagy. Compared with the model group, intervention with sesamin resulted in a decreased volume ratio of intracellular lipid droplets by 51.3% (Figure 2B), an increase in mitochondrial quantity, a restoration of oval and elongated structures, and a clear cristae structure, indicating an improvement on mitochondrial homeostasis (Figure 2C). The increased lysosomes and autophagic lysosomes (Figure 2D) suggest an increase in cellular autophagy levels, conducive to timely removal of damaged mitochondria. The lipid toxicity caused by lipid accumulation may be the main reason for low-level cellular autophagy and premature degradation of damaged mitochondria. Therefore, it could be speculated that sesamin restores mitochondrial function by increasing mitophagy, thereby promoting lipid metabolism and reducing lipid accumulation.

### 3.3 Sesamin improves oxidative stress and mitochondrial damage induced by 9-trans-C18:1 in L02 cells

**Figure 3:**
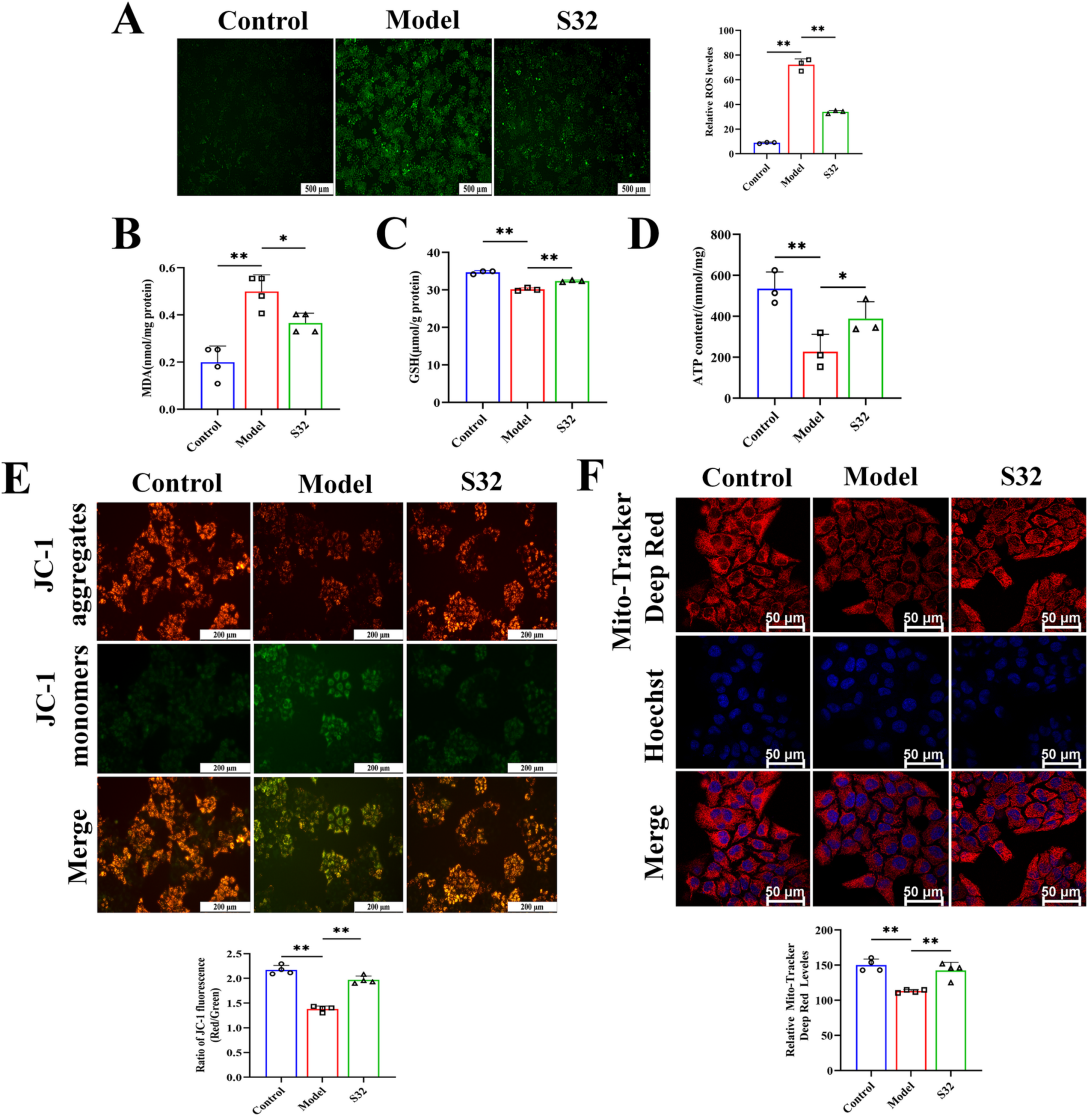
Sesamin improves oxidative stress and mitochondrial impairment induced by 9-trans-C18:1 in L02 cells. **(A)** Semiquantitative measurement of intracellular ROS levels and fluorescence intensity; **(B)** Intracellular MDA levels; **(C)** Intracellular GSH content; **(D)** Intracellular ATP content; **(E)** The level and semiquantitative of Intracellular MMP. **(F)** Mitochondrial far-infrared and semiquantitative analyses; **(A)** Scale bar = 500 μm, **(E)** scale bar = 200 μm, **(F)** scale bar = 50 μm. Control: RPMI 1640 complete culture medium; Model: 500 μM 9-trans-C18:1 + RPMI 1640; S32: 500 μM 9-trans-C18:1 + 32 μM sesamin. All data are represented as means ± SD (*n* = 3–4), *p < 0.05; **p < 0.01.

Subsequently, the impact of sesamin on cellular function was analyzed. ROS serves as an important indicator for cellular oxidative damage. Compared with control, the increased fluorescence intensity in the model group indicates an increase in intracellular oxidative stress level after 9-trans-C18:1 treatment, which could be alleviated by sesamin intervention, demonstrating its mitigating effect on intracellular oxidative stress (Figure 3A). MDA can reflect the degree of lipid peroxidation in the body and indirectly reflect the degree of cell damage. GSH content is an important indicator for body’s antioxidant capacity. Sesamin significantly downregulated the intracellular MDA (Figure 3B), while significantly upregulating the intracellular GSH (Figure 3C), suggesting the role of sesamin in diminishing 9-trans-C18:1 induced oxidative stress level. The weakened mitochondrial MMP acts as the primary indicator for mitochondrial dysfunction. Compared with control, 9-trans-C18:1 treatment resulted in a significant decrease in intracellular MMP, which could be reversed by sesamin (Figure 3E). The mitochondrial far-infrared fluorescence probe can detect cell apoptosis by detecting changes in MMP. The intensity of red fluorescence represents the function of mitochondria. As shown in Figure 3F. sesamin intervention enhanced the function of mitochondria, consistent with the MMP results. Decreased ATP levels usually indicates impaired or decreased mitochondrial function. The significantly reduced ATP levels in the model group were reversed by sesamin, indicating sesamin’s effects on restoring partial mitochondrial function (Figure 3D). The above suggests that sesamin can improve the severe oxidative stress and disrupted mitochondrial homeostasis induced by 9-trans-C18:1 in L02 cells, thereby restoring partial mitochondrial function and diminishing lipid accumulation.

### 3.4 Sesamin activates mitophagy through PINK1/Parkin pathway

**Figure 4:**
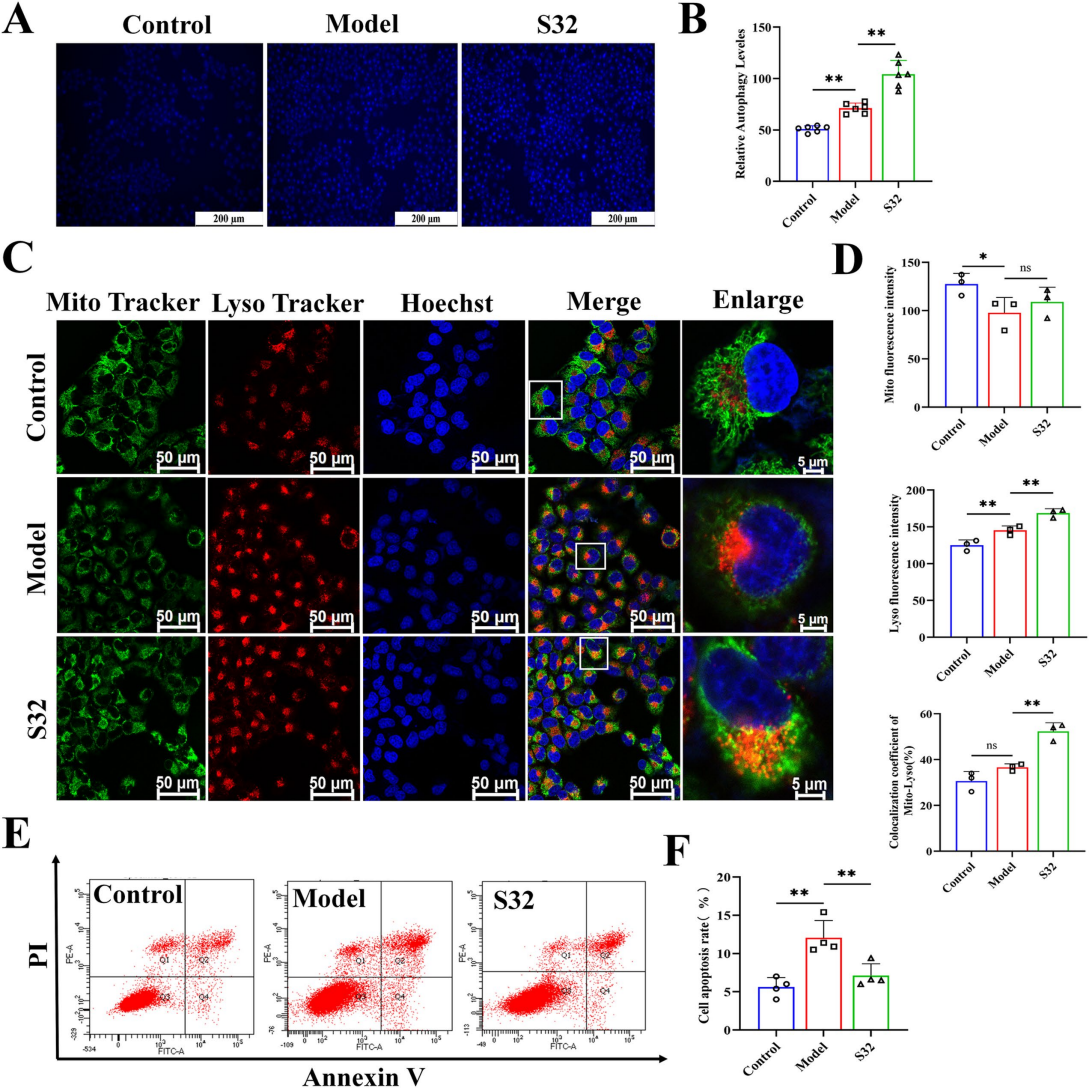
Sesamin activates mitophagy and reduces apoptosis in L02 cells treated by 9-trans-C18:1. **(A,B)** Semiquantitative measurement of intracellular MDC levels and fluorescence intensity; **(C)** Fluorescence intensity photography of Mito-Lyso co-localization; **(D)** Mitochondrial fluorescence intensity, semiquantitative lysosome fluorescence intensity, and Mito-Lyso red green fluorescence co-localization coefficient (*n* ≥ 50 cells per group); **(E,F)** Apoptosis rate and semiquantitative analyses; **(A)** Scale bar = 200 μm, **(C)** scale bar = 50 μm. Control: RPMI 1640 complete culture medium; Model: 500 μM 9-trans-C18:1 + RPMI 1640; S32: 500 μM 9-trans-C18:1 + 32 μM sesamin. All data are represented as means ± SD (*n* = 3–6), *p < 0.05; **p < 0.01.

MDC is one of the most commonly used fluorescent probes for detecting cellular autophagy. Compared with control, autophagy in the model group was enhanced in some cells under 9-trans-C18:1 induction, with autophagy level relatively low, however. Compared with the model group, sesamin improved the cellular autophagy, thereby alleviating the 9-trans-C18:1 induced lipid accumulation to some extent and the degree of cell damage (Figures 4A,B). Next, changes in mitophagy were examined through co-staining with MitoTracker and LysoTracker (Figures 4C,D). Compared with control, the model group showed a significant decrease in mitochondrial fluorescence intensity, indicating that the accumulated lipid droplets significantly reduced mitochondrial homeostasis, in consistence with the TEM observation results (Figure 2). The fluorescence intensity of lysosomes and Mito-Lyso co-localization coefficient in the model group were significantly lower than those in the S32 group, indicating low mitophagy level in the model group. In the S32 group, significant enhanced Lyso fluorescence and Mito-Lyso co-localization coefficient were observed, suggesting that sesamin activated intracellular lysosomes and improved mitochondrial homeostasis by enhancing mitophagy levels, thereby reducing lipid droplet accumulation. As shown in Figures 4E,F, compared with the model group, the apoptosis rate of cells significantly decreased after intervention with sesamin, suggesting that sesamin may alleviate the degree of cell damage by enhancing autophagy levels to clear damaged mitochondria.

**Figure 5:**
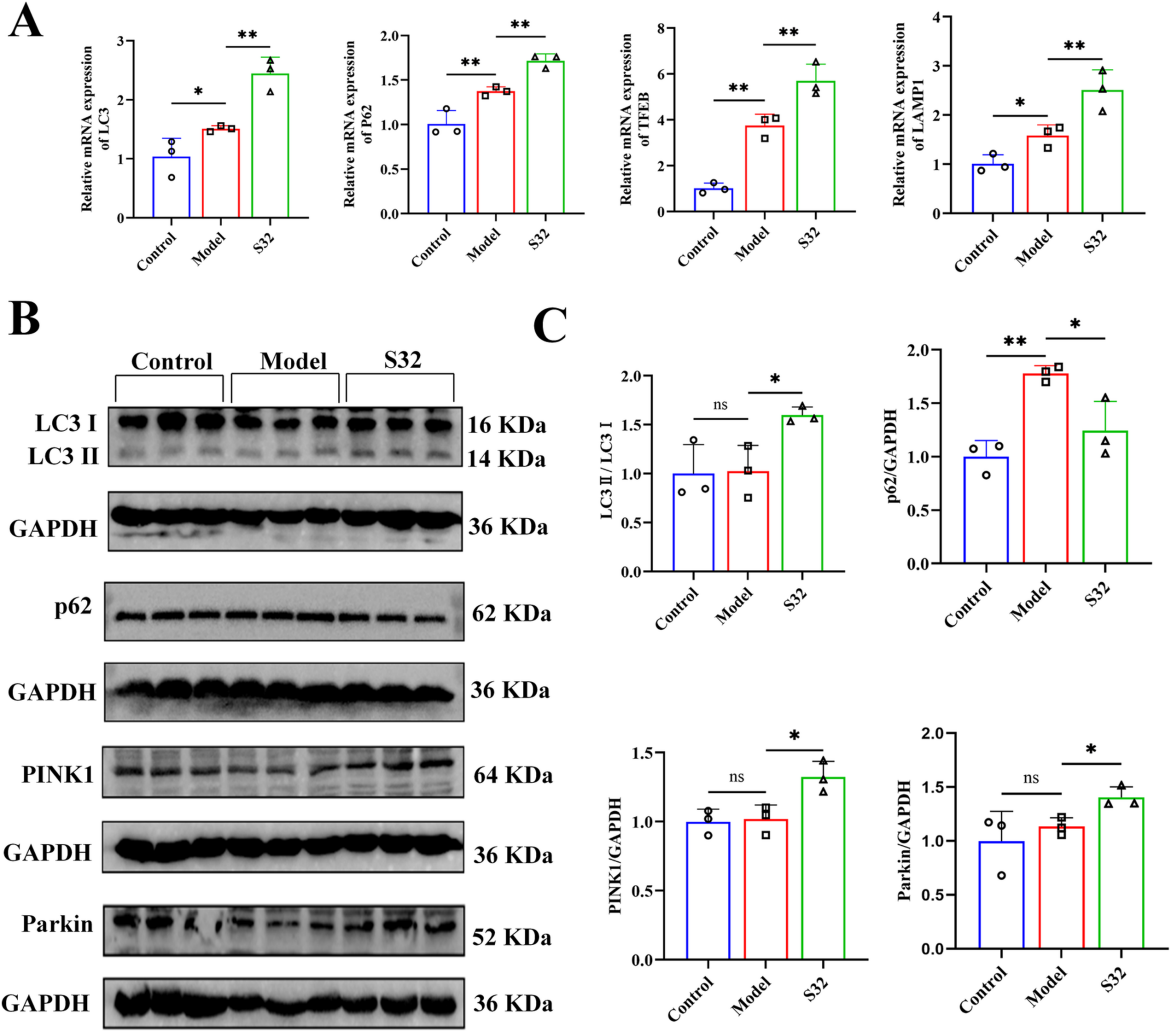
Sesamin enhances mitophagy through the PINK1/Parkin pathway. **(A)** mRNA levels of LC3, p62, TFEB, and LAMP1; **(B,C)** Protein levels of LC3, p62, PINK1, and Parkin proteins. Control: RPMI 1640 complete culture medium; Model: 500 μM 9-trans-C18:1 + RPMI 1640; S32: 500 μM 9-trans-C18:1 + 32 μM sesamin. All data are represented as means ± SD (*n* = 3), *p < 0.05; **p < 0.01.

PINK1/Parkin is a common mitophagy pathway which can be activated by mitochondrial dysfunction (27). Firstly, the mRNA levels of LC3 and p62 were detected by qPCR. Compared with control, the mRNA levels of LC3 and p62 were upregulated in the model group, which could be further upregulated by sesamin (Figure 5A). Next, protein levels of LC3, p62, PINK1, and Parkin were analyzed using Western blot. As shown in Figures 5B,C, in comparison with control, the significantly increased protein levels of p62 suggest low-level autophagy under 9-trans-C18:1, consistent with the results of TEM (Figure 2), MDC (Figures 4A,B), and Mito Lyso co localization (Figures 4C,D). Compared with the model group, sesamin further significantly upregulated the protein levels of LC3, PINK1, and Parkin, while downregulated that of p62, indicating that the PINK1/Parkin pathway was activated, thereby promoting impaired mitophagy and improving mitochondrial homeostasis.

### 3.5 Sesamin activates autophagy process by regulating TFEB nuclear translocation

**Figure 6:**
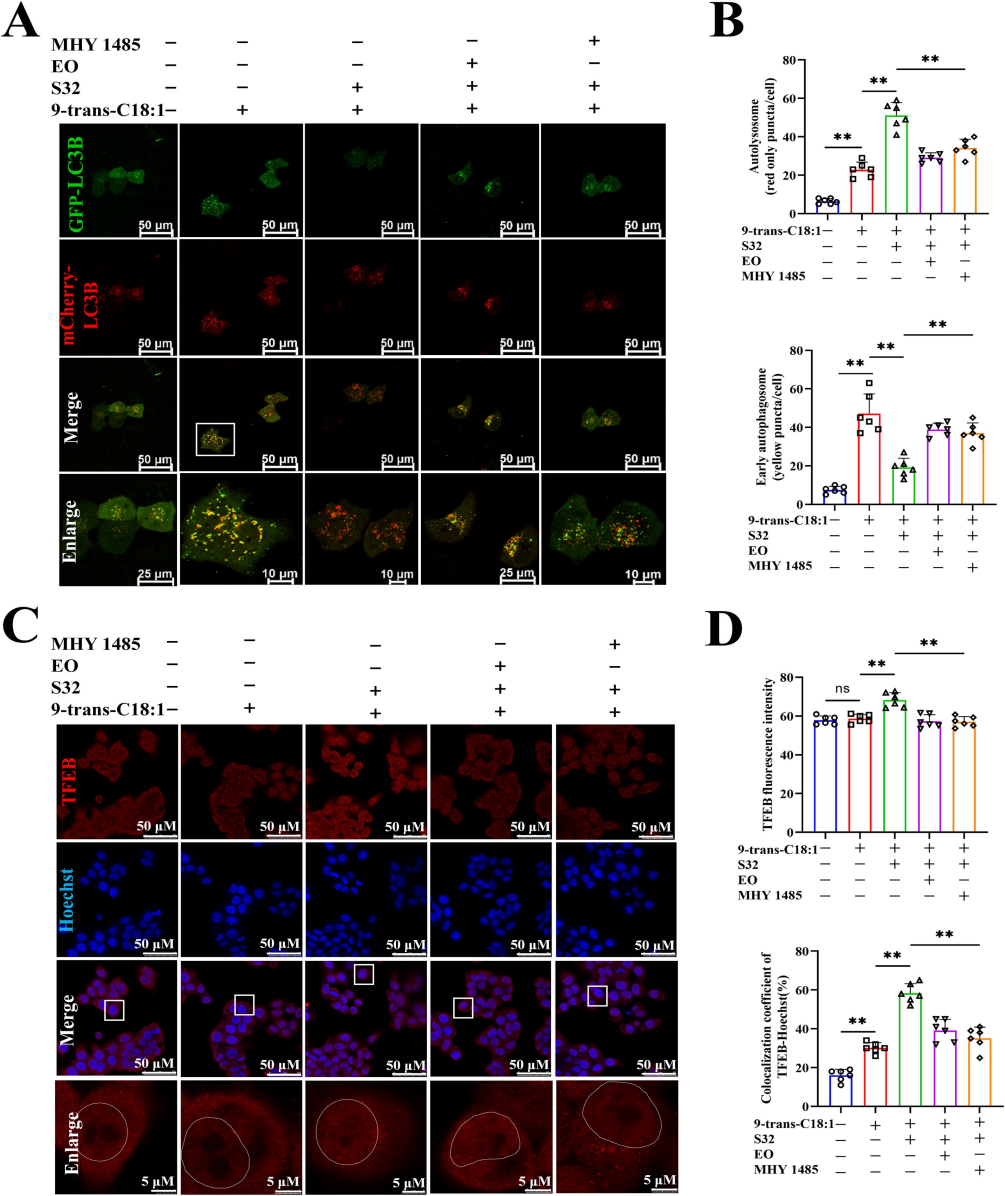
Sesamin elevates autophagic flux in L02 cells treated by 9-trans-C18:1 and promotes the nuclear translocation of TFEB. **(A)** Fluorescence imaging of Ad-mCherry-GFP-LC3B adenovirus transfection; **(B)** Semiquantitative analyses on the number of autophagosomes and early autophagosomes; **(C)** TFEB immunofluorescence staining and photography; **(D)** Semiquantitative for total fluorescence intensity of TFEB and rate of nuclear translocated TFEB (*n* ≥ 50 cells per group); **(A,C)** Scale bar = 50 μm. 9-trans-C18:1: 500 μM 9-trans-C18:1 + RPMI 1640; S32: 500 μM 9-trans-C18:1 + 32 μM sesamin; EO: 10 μM EO+ 500 μM 9-trans-C18:1 + 32 μM sesamin; MHY1485: 2 μM MHY1485+ 500 μM 9-trans-C18:1 + 32 μM sesamin. All data are represented as means ± SD (*n* = 6), *p < 0.05; **p < 0.01.

**Figure 7:**
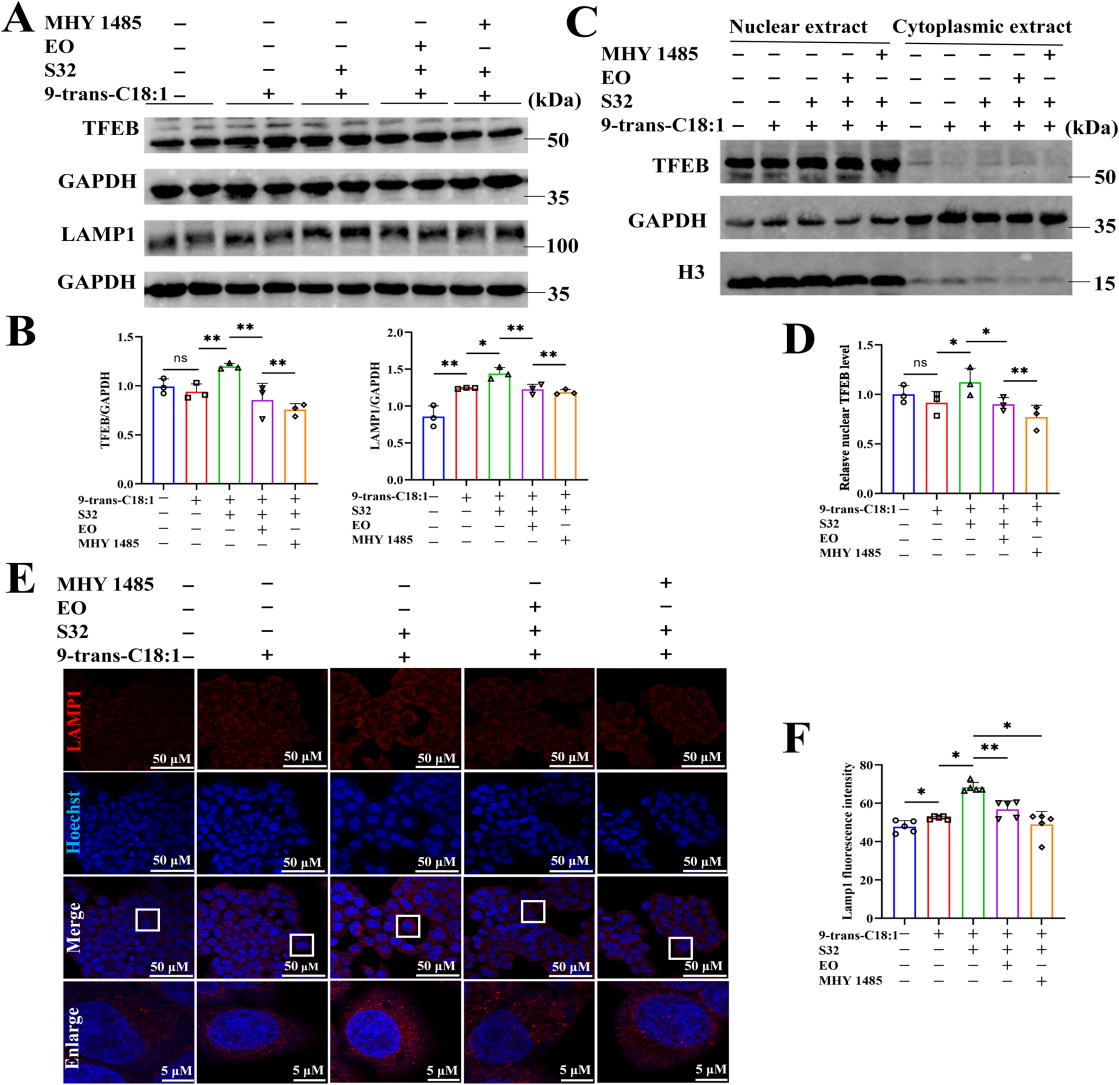
Sesamin activates autophagy by regulating TFEB nuclear translocation. **(A)** Protein levels of TFEB and LAMP1; **(B)** Semiquantitative analyses based on **A**; **(C)** Protein levels of TFEB in nuclear and cytoplasm; **(D)** Relative protein level of TFEB in nuclear; **(E)** LAMP1 immunofluorescence; **(F)** Semiquantitation on LAMP1 immunofluorescence; Scale bar = 50 μm. 9-trans-C18:1: 500 μM 9-trans-C18:1 + RPMI 1640; S32: 500 μM 9-trans-C18:1 + 32 μM sesamin; EO: 10 μM EO+ 500 μM 9-trans-C18:1 + 32 μM sesamin; MHY1485: 2 μM MHY1485+ 500 μM 9-trans-C18:1 + 32 μM sesamin. All data are represented as means ± SD (*n* = 3–5), *p < 0.05, **p < 0.01.

TFEB is activated during lysosomal impairment, which is part of the lysosomal injury response and necessary for subsequent lysosomal recovery, probably promoting lysosomal biosynthesis. The autophagy flux was analyzed through adenovirus transfection to effectively track the fusion process of autophagosomes and lysosomes. The results (Figures 6A,B) showed that the control cells exhibited diffuse yellow fluorescence of mCherry-GFP-LC3B with few autophagosomes and autolysosomes, indicating a non-autophagic state. Cells in the model group presented a large number of yellow spots and few red spots, suggesting that the cells attempted to initiate autophagy to alleviate the impairment caused by 9-trans-C18:1. Compared to the model group, a large number of red spots induced by sesamin suggest an increase in the fusion of autophagosomes and lysosomes and initiated process of autophagosome to autophagosomes to restore partial mitochondrial function, thereby alleviating the oxidative damage induced by 9-trans-C18:1, consistent with the results of TEM (Figure 2). Direct or indirect inhibitor of TFEB, EO or MHY1485, could reverse this trend. The mRNA levels of LAMP1 and TFEB were significantly upregulated before and after sesamin intervention, suggesting the up-regulating effects of both elaidic acid and sesamin on them (Figure 5A). Compared with control, insignificant changes in the overall fluorescence intensity of TFEB but significantly elevated nuclear translocation rate in the model group were observed (Figures 6C,D), indicating a role of 9-trans-C18:1 in dephosphorylating TFEB and inducing nuclear translocation partially. In comparison with the model group, the overall fluorescence intensity of TFEB was significantly enhanced and the translocation rate was significantly increased after intervention with sesamin, suggesting the activating effect of sesamin on TFEB translocation to initiate autophagy. EO or MHY1485 counteracted this effect. Compared with the model group, sesamin significantly upregulated the overall protein levels of TFEB (Figures 7A,B) and that in the nucleus (Figures 7C,D), with that in the cytoplasm declined. EO or MHY1485 reversed this trend, consistent with the immunofluorescence results (Figures 6C,D). Therefore, it could be speculated that sesamin promotes autophagy via regulating the translocation of TFEB.

The immunofluorescence staining of LAMP1 (Figures 7E,F) found that compared with control, the fluorescence intensity of LAMP1 in the model group was enhanced, indicating increased lysosomes to a certain extent after 9-trans-C18:1 treatment. The fluorescence intensity of LAMP1 was significantly enhanced by sesamin, which could be reversed by EO or MHY1485. This suggests that sesamin augments the count of lysosomes, thereby initiating autophagy and alleviating cellular impairment induced by 9-trans-C18:1. The Western blotting (Figures 7A,B) results exhibited upregulate the protein level of LAMP1 by sesamin, consistent with the immunofluorescence results.

**Figure 8:**
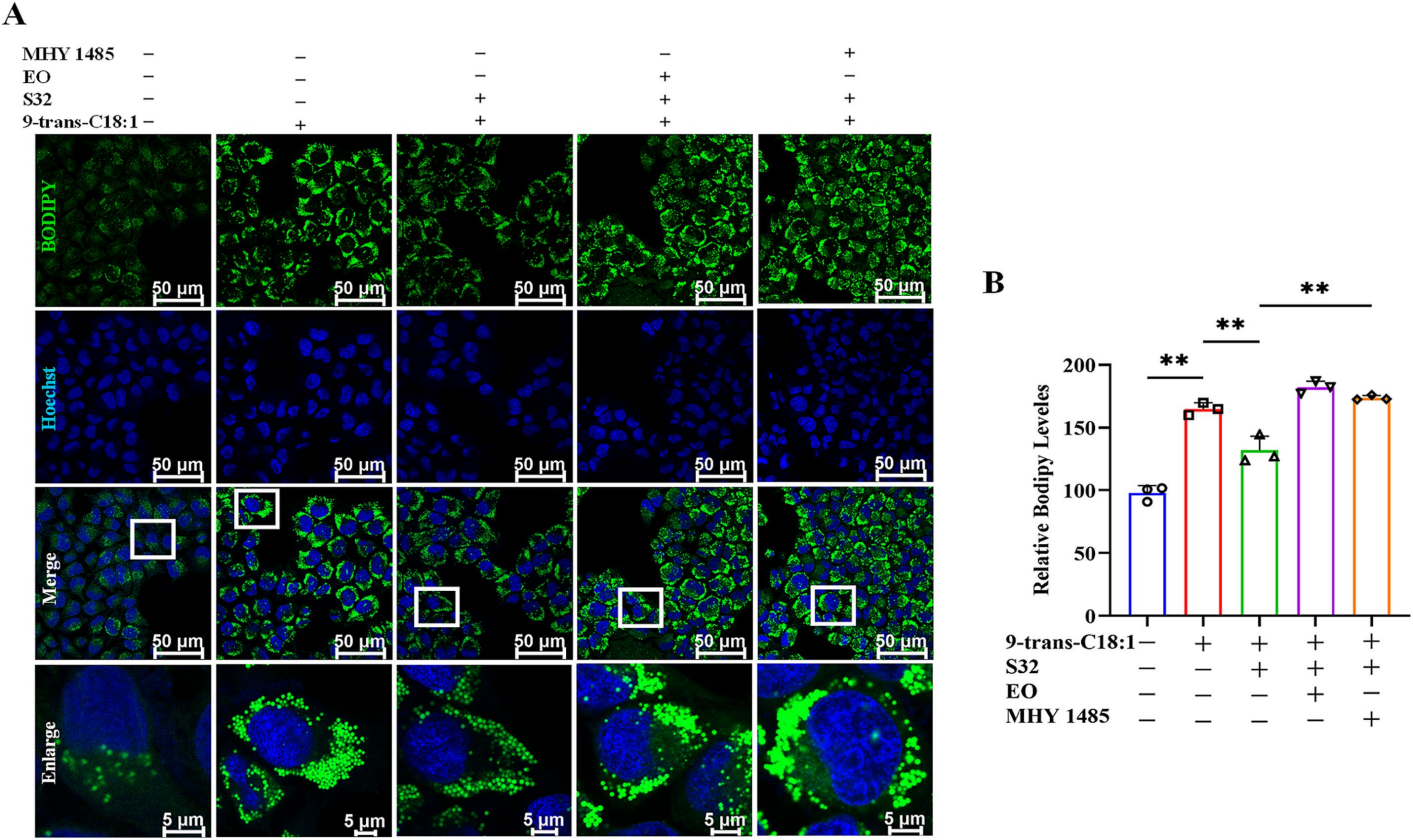
Sesamin mitigates lipid accumulation in L02 cells by regulating TFEB nuclear translocation. **(A)** Lipid droplet fluorescence staining by BODIPY 493/503; **(B)** Semiquantitative analyses based on **A**. Scale bar = 50 μm. 9-trans-C18:1: 500 μM 9-trans-C18:1 + RPMI 1640; S32: 500 μM 9-trans-C18:1 + 32 μM sesamin; EO: 10 μM EO+ 500 μM 9-trans-C18:1 + 32 μM sesamin; MHY1485: 2 μM MHY1485+ 500 μM 9-trans-C18:1 + 32 μM sesamin. All data are represented as means ± SD (*n* = 3), *p < 0.05; **p < 0.01.

BODIPY 493/503 probe can be used to characterize intracellular lipid droplets. The results (Figures 8A,B) showed that compared to control, the green fluorescence of the model group significantly increased. The fluorescence intensity was significantly diminished by sesamin. However, EO or MHY1485 could reverse the effect of sesamin. This suggests that sesamin may alleviate lipid accumulation induced by 9-trans-C18:1 through increasing mitophagy, thereby restoring partial mitochondrial function and mitigating the cellular impairment. As aforementioned, sesamin may activate the autophagy pathway regulated by TFEB via increasing the number of lysosomes, accelerating the process of autophagosome to autophagosomes to remove damaged mitochondria, thereby relieving the degree of cell damage induced by 9-trans-C18:1 and reducing lipid accumulation in L02 cells.

## 4 Discussion

In the present work, the mitigative effects of sesamin on steatosis induced by 9-trans-C18:1 in L02 cells were investigated. The results showed that sesamin significantly reduced lipid accumulation in L02 cells by inhibiting oxidative stress, restoring mitochondrial morphology and function, and reducing cell apoptosis rate. Further analyses exhibited the involvement of autophagy in this process: sesamin may increase autophagy flux, promote nuclear translocation of TFEB and the expression of its target protein LAMP1, activate the autophagy-lysosome pathway to induce protective autophagy, thereby alleviating cell damage caused by 9-trans-C18:1 and reducing lipid accumulation in L02 cells.

In recent years, more and more attention has been focused on dietary ingredients or natural products that can reduce liver steatosis and alleviate NAFLD (28). Sesamin can inhibit fatty acid synthesis, induce fatty acid beta oxidation, and promote the expression of genes related to cholesterol efflux and catabolism by activating the AMPK and PPARα signaling pathways, thereby alleviating lipid accumulation during NAFLD and preventing liver steatosis (11, 14, 29, 30). Pham et al. reported that sesamin reduces palmitic acid-induced lipid toxicity in human hepatocellular carcinoma cell line (HepG2) and promotes liver lipid metabolism by activating the estrogen receptor alpha/calcium/calmodulin-dependent protein kinase *β*/AMPK signaling pathway (12). In addition, it was found that sesamin can improve liver steatosis and fibrosis in mice with NASH (31, 32). Similarly, the present study found that sesamin can significantly reduce lipid accumulation induced by 9-trans-C18:1 in L02 cells, improve cell morphology and physiological function. It has been found that free fatty acid causes oxidative stress in hepatocytes, leading to lipid toxicity and hepatic steatosis. Sesamin can regulate lipid disorders by reducing lipid accumulation and inflammation in NAFLD (33). Here, it could also be found that sesamin significantly reduced intracellular ROS levels, apoptosis degree, and MDA, as well as enhancing GSH release levels. Sesamin indeed exerts antioxidant activity and protective effects on lipid accumulation in hepatocytes, while the underlying mechanisms needs further clarification.

Autophagy, a relatively conserved process in evolution, can transport cellular contents to lysosomes for degradation (34). Excessive TG and FFA in NAFLD patients can induce oxidative stress, leading to impaired autophagy (35, 36). Impaired autophagy can lead to abnormal lipid accumulation in mouse liver and *in vitro* cultured hepatocytes (37), and blocking autophagy can also cause excessive lipid accumulation in the liver (38). Restoring autophagy through genetic or chemotherapy can alleviate hepatic steatosis in obese mice (39, 40). Therefore, impaired autophagy can lead to the occurrence and progression of NAFLD (41). Enhancing autophagy is considered one of the most effective means to alleviate lipid accumulation and has significant implications for the treatment of NAFLD. In the present work, TEM results suggest that sesamin may alleviate lipid accumulation in L02 cells induced by 9-trans-C18:1 through activating mitophagy. The lipid toxicity caused by lipid accumulation may be the main reason for low-level autophagy and premature degradation of impaired mitochondria. Mitochondrial dysfunction can lead to lipid deposition in hepatocytes, causing hepatocytes damage and thus participating in the progression of NAFLD. Some scholars also believe that NAFLD is a mitochondrial disease (42). Mitophagy acts as one of the important means to remove dysfunctional mitochondria prior to activating apoptosis, by breaking down and reusing impaired mitochondria to maintain their normal structural stability (43, 44). Therefore, sesamin might restore mitochondrial function in 9-trans-C18:1 treated L02 cells by increasing mitophagy, thereby promoting lipid metabolism and reducing lipid accumulation. The accumulated ROS enhances mitochondrial impairment, which is one of the factors triggering mitophagy (45). Park et al. found that the lipid toxicity induced by saturated fatty acids is mediated by excessive production of ROS, related to mitochondrial impairment (46). In addition, Liu et al. believe that declined MMP is a hallmark of mitochondrial dysfunction (47). Consistently, 9-trans-C18:1 treatment enhanced intracellular ROS production while reducing MMP, accompanied by inhibited ATP generation. Meanwhile, the far-infrared fluorescence representing the physiological activity of mitochondria also significantly weakened, indicating the occurrence of mitochondrial dysfunction. Interestingly, this process can be reversed by sesamin. After mitophagy was activated by various stimuli such as oxidative stress, levels of PINK1 and Parkin, p62 and autophagy related 16 like (Atg16L) protein, along with the autophagy marker LC3II/LC3I were correspondingly upregulated (48). Quercetin alleviates mitochondrial impairment and oxidative stress by enhancing Frataxin mediated PINK1/Parkin dependent mitophagy, thereby preventing liver lipid accumulation (49). Therefore, activating mitophagy may possess therapeutic potential in reducing hepatic steatosis.

PINK1/Parkin dependent mitophagy is mainly involved in recognizing and clearing damaged mitochondria. Normally, PINK1 enters the mitochondrial inner membrane under the guidance of mitochondrial targeting sequences, and is subsequently cleaved by proteases on the inner membrane and released into the cytoplasm for hydrolysis. MMP decreases in the case of mitochondrial impairment, leading to the accumulation of PINK1 on the outer membrane of mitochondria. At the same time, Parkin undergoes phosphorylation and activation, selectively recruiting to damaged mitochondria. During this process, p62 is also recruited to mitochondria and then binds to LC3, mediating mitophagy (50, 51). Knockout of mitophagy genes of Parkin and ATG7 exacerbated myocardial injury in high-fat induced mice (52).

In the progression from NAFLD to NASH, the clearance of damaged mitochondria, inhibition of inflammatory mediators, and increased fatty acid oxidation are all associated with mitophagy (53). Overexpression of PINK1 can effectively restore mitophagy flux in aging macrophages (48). Decreased PINK1 in aging lungs leads to impaired mitophagy, promoting pulmonary fibrosis in young mice (54). Similarly, in this study, sesamin enhanced mitophagy and significantly reduced cell apoptosis rate. At the same time, sesamin significantly upregulated the ratio of LC3II/LC3I and protein levels of PINK1 and Parkin while downregulated p62 in L02 cells treated by 9-trans-C18:1, suggesting that sesamin indeed activates autophagy flux through the PINK1/Parkin pathway. Autophagy is a dynamic process, autophagosomes fuse with lysosomes and are subsequently degraded and cleared by lysosomes at the final stage of autophagic flux (55). Defects in lysosomal biogenesis and clearance inhibit autophagic flux, leading to lipid accumulation during the development of NAFLD (10). It has found that lysosomal biosynthesis in hepatocytes can be disrupted by high-fat diet (HFD), which is related to lipid metabolism (56). HFD induced hepatic steatosis can be alleviated by increasing autophagy flux (57). EO is a potent TFEB inhibitor and an effective and safe autophagy inhibitor (58). In addition, TFEB activity is also negatively regulated by the mechanistic target of rapamycin kinase complex 1 (mTORC1) (59), and mechanistic target of rapamycin kinase (mTOR) activation leads to TFEB phosphorylation in the cytoplasm and loss of activity. MHY1485, a mTOR agonist, can indirectly inhibit TFEB activation translocation to the nucleus (60). In this study, sesamin indeed enhanced autophagic flux employing EO and MHY1485. In addition, L02 cells initiated the process of autophagosome to autophagosome to alleviate oxidative damage induced by 9-trans-C18:1, thereby improving normal cellular morphology and function, restoring mitochondrial function partially, and alleviating cellular damage. EO and MHY1485 significantly reversed the increase in autophagy flux induced by sesamin. Therefore, it could be inferred that sesamin may activate autophagy inhibited by 9-trans-C18:1 in an autophagy-lysosome pathway dependent manner. Similarly, autophagy activation is beneficial and crucial for sesamin mediated alleviation of oxidative damage and lipid metabolism disorder induced by 9-trans-C18:1 in L02 cells. It has also been demonstrated that sesamin can activate the autophagy-lysosome pathway to alleviate lipid accumulation due to 9-trans-C18:1 treatment in L02 cells. However, further clarification is needed on whether sesamin activates the PINK1/Parkin autophagy pathway through TFEB.

TFEB has been identified as the primary regulatory factor for autophagy and lysosomal biogenesis. Under normal conditions, TFEB exists in the cytoplasm in an inactive phosphorylated form, while fasting and stress conditions induce its dephosphorylation into the nucleus. Nuclear translocated TFEB can promote lysosomal biogenesis and substrate degradation (61), and induce expression of genes involved in lysosomal biogenesis (62). TFEB can promote the expression of genes related to lipid metabolism. In NAFLD patients, hepatic steatosis leads to TFEB phosphorylation mainly occurring in the cytoplasm. Therefore, TFEB activation may be an important marker for determining the degree of hepatic steatosis in NAFLD patients and associated with decreased autophagy activity (61). Overexpression of TFEB promotes autophagosome lysosome biogenesis, thereby inhibiting ethanol induced liver injury and steatosis in mice (63). Elevated levels of TFEB in the liver upregulates genes encoding lipid metabolism to oxidize FFA in mitochondria (64). The activation of TFEB-growth differentiation factor-15 (GDF15) in macrophages can regulate metabolic disorders such as obesity induced by HFD (65). The deficiency of TFEB in hepatocytes leads to a decrease in lipid metabolism, exacerbating metabolic diseases such as HFD induced hepatic steatosis and insulin resistance (66). There are also studies revealing that TFEB promotes nuclear translocation by activating the AMPK pathway and alleviates hepatic steatosis in mice fed with HFD (67). Here, it could be learned that sesamin can induce TFEB nuclear translocation and up-regulate its downstream target protein LAMP 1 to activate autophagy flux, and promote lysosomal biosynthesis. Therefore, sesamin probably promotes TFEB nuclear translocation and activates autophagosome-lysosome processes to reduce lipid accumulation in hepatocytes. Interestingly, TFEB activity may also be regulated by several key factors involved in autophagy, such as PINK1 and Parkin (68). Consistent with this, after treatment with sesamin, the mRNA levels of LC3 and p62, along with the ratio of protein LC3II/LC3I, significantly increased in L02 cells treated by 9-trans-C18:1. At the same time, levels of PINK1 and Parkin were enhanced, with p62 downregulated. Finally, it was found through BODIPY fluorescence staining that the lipid-lowering effect of sesamin can be reversed by EO or MHY1485. This suggests that sesamin may alleviate lipid accumulation via promoting intracellular mitophagy levels in a high-lipid *in vitro* model induced by 9-trans-C18:1, thereby restoring partial mitochondrial function and alleviating cell damage. Therefore, sesamin exerts its antioxidant stress and reduces lipid accumulation in cells by activating autophagy regulated by TFEB. However, the exact mechanism by which TFEB regulates autophagy in L02 cells still needs further clarification.

This study revealed the mechanism by which sesamin exerts antioxidant stress and reduces lipid accumulation in L02 cells, and identified the important role of transcription factor TFEB in the autophagosome-lysosome pathway. However, our study also has some limitations. It has been reported that curcumin can induce Parkin dependent autophagy by activating AMPK and subsequent TFEB nuclear translocation, improve oxidative stress, enhance intestinal barrier function, and mitochondrial function (44). Therefore, the specific mechanism by which sesamin induces TFEB to regulate autophagy in L02 cells needs further clarification. In addition, it is necessary to explore other mechanisms by which sesamin induces autophagy in L02 cells. Finally, this study was only validated in an in vitro cell model and also needs to be validated *in vivo*. In our future work, it is necessary to explore more comprehensively underlying mechanisms by which sesamin regulates lipid metabolism.

## 5 Conclusion

Sesamin restores impaired mitochondrial morphology and function by activating the TFEB pathway, and significantly alleviates lipid accumulation induced by 9-trans-C18:1 in L02 cells, which may exert preventive or protective effects on hepatic steatosis. In the future, it is necessary to further explore the protective effects of sesamin on steatosis through multiple omics methods such as transcriptomics, proteomics, and metabolomics.

## Data Availability

The original contributions presented in the study are included in the article/supplementary material, further inquiries can be directed to the corresponding author/s.
